# Effect of incubation on freezability of cholesterol-loaded cyclodextrin treated buffalo (*Bubalus bubalis*) spermatozoa

**DOI:** 10.14202/vetworld.2015.182-185

**Published:** 2016-02-18

**Authors:** S. A. Lone, J. K. Prasad, S. K. Ghosh, G. K. Das, B. Balamurugan, R. Katiyar, M. R. Verma

**Affiliations:** 1 Division of Animal Reproduction, Gynecology & Obstetrics, National Dairy Research Institute, Karnal - 132001, Haryana, India; 2 Germplasm Centre, Division of Animal Reproduction, Indian Veterinary Research Institute, Izatnagar, Bareilly - 243122, Uttar Pradesh, India; 3 Division of Livestock Economics and Statistics, Indian Veterinary Research Institute, Izatnagar, Bareilly - 243122, Uttar Pradesh, India

**Keywords:** buffalo spermatozoa, cholesterol loaded cyclodextrin, incubation

## Abstract

**Aim:**

The aim of this study was to investigate the effect of incubation on freezability of cholesterol loaded cyclodextrin (CLC) treated buffalo spermatozoa.

**Materials and Methods:**

Semen samples with mass motility of 3+ and greater, collected from Murrah buffalo bulls were utilized. Immediately after collection, four equal groups of semen sample were made. Group I was kept as control and diluted with Tris upto concentration of 60×10^6^ sperm/ml, where as Groups II, III, and IV were treated with CLC at 3 mg/120× 10^6^ spermatozoa, incubated at 37°C for action of CLC for 10, 15 and 20 min, respectively, and diluted with tris upto concentration of 60×10^6^ sperm/ml. All groups were subjected to equilibration and freezing. The evaluation of semen samples from all groups was carried out at fresh, pre-freeze and post-thaw stage for progressive motility, viability and hypo-osmotic swelling response (HOS response).

**Results:**

At the pre-freeze stage, significantly (p<0.05) higher percentage of progressive motility and viability was observed in treatment groups as compared to control with no significant difference among treatment groups. HOS response was significantly (p<0.05) higher in treatment groups as compared to control at pre-freeze stage. At post-thaw stage, significantly (p<0.05) higher percentage of progressive motility, viability and HOS response was recorded in Group II as compared to control and other treatment groups (III and IV). Group II retained significant post-thaw motility and viability at various post-thaw incubation periods.

**Conclusion:**

Incubation period of 10 min for CLC treated buffalo spermatozoa yielded significantly higher results in terms of freezability as compared to incubation for 15 and 20 min.

## Introduction

Plasma membrane integrity is essential for spermatozoa to protect them from harmful effects of cryopreservation. Adding cholesterol or its analogs to the medium reduces capacitation process [1]. Due to hydrophobic nature of cholesterol, it is insoluble in aqueous semen diluents. Cyclodextrins, which are obtained by enzymatic degradation of starch, are capable of inserting cholesterol into cell membranes due to the presence of an internal hydrophobic core, in addition to an external hydrophilic face [2].

Addition of CLC to semen significantly increases progressive motility, viability, acrosomal integrity [3,4], and hypo-osmotic swelling (HOS) responsive spermatozoa [3]. CLC improves *in vitro* fertilizing ability and reduces ultrastructural damages to spermatozoa plasma membrane [4]. CLC has been used for modification of cholesterol content in buffalo spermatozoa [5].

After treating the spermatozoa with CLC, some incubation period is needed for transfer of cholesterol into sperm plasma membrane [6]. Incubation of CLC treated spermatozoa at 22°C or 37°C for 15, 30 and 60 min have revealed similar beneficial effects in terms of cryopreservation of cattle bull semen [7]. However, no study has been conducted regarding the effect of different incubation periods on freezability of CLC treated buffalo spermatozoa.

The aim of this study was to investigate the effect of incubation on freezability of CLC treated buffalo spermatozoa.

## Materials and Methods

### Ethical approval

No ethical approval was necessary to pursue this research work.

### Climatic conditions and experimental animals

Geographical location of Bareilly is at an altitude of 169 m above mean sea level and at the latitude of 28.22°N and longitude of 79.22°E. Bareilly is known to have a moderate climate. Summer temperature goes up to 40°C, where as winter temperature goes down up to 8°C. The rainy season starts in June and extends up to September with humid and warm conditions. Three healthy breeding buffalo bulls maintained at Germplasm Center, Division of Animal Reproduction, Indian Veterinary Research Institute (IVRI), Izatnagar, Bareilly were utilized for the study. These bulls were kept under identical feeding and management conditions during the entire course of the investigation.

### Chemicals

All chemicals were reagent grade and purchased from Sigma-Aldrich (St. Louis, MO, USA).

### CLC preparation

Methyl-β-CLC was loaded with cholesterol as described [7]. Briefly, 200 mg of cholesterol was dissolved in 1 ml of chloroform in a glass tube. In the second glass tube, 1 g of methyl-β-cyclodextrin was dissolved in 2 ml of methanol. A 0.45 ml aliquot of the cholesterol solution was added to the cyclodextrin solution, and the mixture was stirred until the combined solution appeared clear. This was followed by pouring of the mixture into a glass petri-dish and removing of solvents using a stream of nitrogen gas. The resulting crystals were allowed to dry for an additional 24 h and then were removed from the dish and stored in a glass container at 22°C. A working solution of CLC was prepared by adding 50 mg of CLC to 1 ml of tris diluent at 37°C and mixing the solution briefly using a vortex mixer.

### Collection of semen and its processing

Semen was collected using an artificial vagina as per the standard method. A total of 24 ejaculates, eight from each bull (8 × 3 = 24) were collected. Only ejaculates with mass motility ≥3+ were used in the study. Immediately after collection of semen, each ejaculate was divided into four groups. Group I: Control (without addition of CLC and diluted with tris-egg yolk-glycerol dilutor up to 60×10^6^ spermatozoa/ml. In Groups II, III and IV, CLC was added at 3mg/120×10^6^ spermatozoa and were incubated at 37°C for 10, 15 and 20 min, respectively, for entry of cholesterol into sperm plasma membrane. After incubation each sample was diluted with tris-egg yolk-glycerol dilutor up to 60×10^6^ spermatozoa/ml. Initial progressive motility, viability and HOS response were recorded in each group.

### Semen freezing and its evaluation

French midi straws (0.5 ml) were filled with the extended semen samples, sealed with polyvinyl alcohol powder and kept for 3 h at 5°C for equilibration. After equilibration, straws were kept in automatic programable biological cell freezer (IMV technology, France) until temperature of straws reached −145°C. Then, straws were plunged into liquid nitrogen (−196°C) for storage. Semen samples were evaluated at pre-freeze and post-thaw stage for progressive motility, viability and HOS response. The viability of spermatozoa was assessed using Eosin-Nigrosin stain described by Campbell *et al*. [8].

### Statistical analysis

Data were statistically analyzed by one-way ANOVA and results were expressed as mean ± standard error. Means were compared using Tukey's multiple comparison test. The statistical package of Graph pad prism, San Diego, USA was used for analyzing the data.

## Results and Discussion

### Effect of incubation on individual progressive motility, viability and HOS response at fresh stage

The initial progressive motility of a semen sample gives a good indication of the fertility of the bull and ability of spermatozoa to withstand the stress of the cryopreservation process. The percent individual progressive motility of spermatozoa was 82.29±0.67, 82.50±0.65, 82.50±0.65 and 82.29±0.67, respectively, in Groups I, II, III and IV (Table-1).The percent individual progressive motility was comparable to the values reported by workers [5,9] but higher than the values reported by Sannat *et al*. and Bhakat *et al*. [10,11]. The viability of spermatozoa in a semen sample is significantly and positively correlated with initial motility, post-thaw motility, and fertility of spermatozoa. Percentage of viable spermatozoa was 87.45±0.66, 87.79±0.68, 87.16±0.69, and 87.16±0.65, respectively, in Group I, II, III and IV (Table-1). The percentage of viable spermatozoa was comparable to values reported by Ramteke [12], higher than the values reported by Rajoriya et al. [5] but lower than the values of Shukla and Misra [13].The difference may be attributed to the various factors that affect motility and viability like bull's age, season, frequency of collection and sexual excitement before semen collection.

**Table-1:** Mean±SE of effect of incubation on progressive motility, viability and HOS response at fresh stage.

Seminal attribute	Group I	Group II	Group III	Group IV
Progressive motility (%)	82.29±0.67	82.50±0.65	82.50±0.65	82.29±0.67
Viability (%)	87.45±0.66	87.79±0.68	87.16±0.69	87.16±0.65
HOS response (%)	77.16±0.70	77.54±0.71	77.33±0.70	77.25±0.70

SE=Standard error, HOS=Hypo-osmotic swelling

Hypo-osmotic swollen spermatozoa (%) were 77.16±0.70, 77.54±0.71, 77.33±0.70 and 77.25±0.70, respectively, in Group I, II and III and IV (Table-1). No significant difference in mean values of hypo-osmotic swollen spermatozoa (%) was observed among Groups I, II, III and IV. The percent HOS positive spermatozoa in our study was higher than the values reported by Kumar [3] and Kadirvel *et al*. [14] but lower than that reported by Rajoriya *et al*. [15]. The higher percentage of HOS positive spermatozoa in our study indicates higher percentage of membrane intact spermatozoa.

### Effect of incubation on individual progressive motility, viability and HOS response at pre-freeze and post-thaw stage

The percent individual progressive motility at pre-freeze was significantly (p<0.05) higher in treatment groups as compared to control group. No significant difference in percent individual progressive motility was observed among treatment Groups (II, III, and IV). At post-thaw stage, group II had highest (59.58±0.41) individual progressive motility compared to other three groups. A significantly (p<0.05) higher percentage of progressively motile spermatozoa in Group II was observed as compared to Group I (control). The post-thaw progressive motility was significantly (p<0.05) higher in Group II as compared to Groups (III and IV). No significant difference was observed in mean values of progressively motile spermatozoa among Groups (III and IV). In our study about 4% increase in individual progressive motility in Group II was recorded as compared to Group III (Table-2). At pre-freeze stage, percent live spermatozoa was significantly (p<0.05) higher in the treatment groups as compared to control group (Group I). No significant difference was observed in mean percent live spermatozoa among treatment groups. The mean post-thaw percent live spermatozoa was higher in treatment groups with Group II having significantly (p<0.05) higher mean post-thaw percent live spermatozoa compared to Group I (control). Percent mean sperm viability was significantly (p<0.05) higher in Group II as compared to Groups (III and IV). However, no significant difference in post-thaw percent live spermatozoa was observed between Groups III and IV. On an average 3% increase in percentage of live spermatozoa was observed in Group II as compared to Group III (Table-2). Our study revealed that at pre-freeze stage, incubation for 10, 15 and 20 min yielded no significant difference in terms of progressive motility and viability of buffalo spermatozoa among treatment groups. However at post-thaw stage, 10 min incubation revealed significant results in terms of freezability of buffalo spermatozoa as compared to incubation at higher 15 and 20 min.

**Table-2:** Mean±SE of effect of incubation on progressive motility, viability and HOS response at pre-freeze and post-thaw stage of buffalo spermatozoa.

Groups	Progressive motility (%)		Viability (%)		HOS response (%)	
		
	Pre-freeze	Post-thaw	Pre-freeze	Post-thaw	Pre-freeze	Post-thaw
Group I	71.25±0.68^b^	50.58±0.39^c^	75.29±0.64^b^	55.95±0.39^c^	66.70±0.64^b^	50.20±1.13^c^
Group II	76.45±0.64^a^	59.58±0.41^a^	81.29±0.61^a^	64.62±0.35^a^	73.20±0.71^a^	60.08±1.09^a^
Group III	75.83±0.62^a^	55.62±0.42^b^	79.87±0.63^a^	61.45±0.34^b^	71.62±0.70^a^	57.75±1.18^b^
Group IV	74.58±0.77^a^	54.74±0.35^b^	78.95±0.65^a^	60.25±0.31^b^	70.25±0.70^a^	55.58±1.14^b^

Values bearing different superscripts in column (a, b and c) differ significantly at 5%(p<0.05). SE=Standard error, HOS=Hypo-osmotic swelling

At pre-freeze stage, percent HOS response was significantly (p<0.05) higher in treatment groups as compared to Group I (control). No significant difference in HOS response was observed between Groups III and IV. At post-thaw stage, percent HOS response was significantly higher in Group II than all other groups. Among treatment groups, the HOS response was significantly (p<0.05) higher in Group II as compared to Groups III and IV. No significant difference in HOS response was observed between Groups III and IV at pre-freeze and post-thaw stage. At post-thaw stage, there was an increase of about 3% HOS responsive spermatozoa in Group II as compared to Group III (Table-2).

This clearly indicated 10 min incubation has beneficial effect on membrane integrity. No literature could be traced to compare our finding in the case of buffalo semen.

All four groups were also subjected to post-thaw incubation test to observe sustainability of post-thaw motility and viability after a prolonged duration of thawing (Tables-3 and 4). Our study revealed a significant reduction in post-thaw motility and viability from 0 min to 120 min. Group II withstands satisfactory post-thaw motility (50.00%) even after 30 min of post-thaw incubation period (Table-3). In Group II, reduction in post-thaw livability was significantly less as compared to Groups I, III and IV (Table-4). Group II showed satisfactory viability (55.66%) at 30 min of post-thaw incubation period (Table-4). In the present study, results in terms of post-thaw incubation test with respect to post-thaw motility and viability were significantly (p<0.01) better in Group II as compared to other three groups.

**Table-3:** Mean±SE of effect of incubation on post-thaw motility at various post-thaw incubation periods.

Time (min)	Group I (control)	Group II	Group III	Group IV
0	50.58±0.39^C^	59.58±0.41^A^	55.62±0.42^B^	54.74±0.35^B^
15	41.25±0.35^c^	55.20±0.31^a^	50.20±0.41^b^	50.00±0.37^b^
30	31.87±0.33^c^	50.00±0.33^a^	44.58±0.44^b^	43.25±0.41^b^
45	23.54±0.35^d^	45.00±0.33^a^	38.12±0.44^b^	36.91±0.41^c^
60	15.20±0.43^d^	40.00±0.33^a^	32.87±0.37^b^	30.58±0.46^c^
120	6.66±0.27^d^	26.87±0.44^a^	19.16±0.32^b^	16.45±0.38^c^

Values bearing different superscripts in upper case letters in a row differ significantly at 5% (p<0.05) and in lower case letters in a row differ significantly at 1% (p≤0.01). SE=Standard error

**Table-4:** Mean±SE of effect of incubation on post-thaw viability at various post-thaw incubation periods.

Time (min)	Group I (control)	Group II	Group III	Group IV
0	55.95±0.396^C^	64.62±0.359^A^	61.45±0.347^B^	60.25±0.319^B^
15	47.20±0.365^c^	59.87±0.317^a^	55.41±0.378^b^	54.00±0.308^b^
30	37.66±0.371^d^	55.08±0.293^a^	49.58±0.391^b^	47.00±0.285^c^
45	29.20±0.384^d^	49.95±0.328^a^	43.37±0.400^b^	40.50±0.426^c^
60	20.59±0.397^d^	44.79±0.307^a^	37.16±0.366^b^	33.54±0.465^c^
120	11.37±0.262^d^	32.33±0.426^a^	23.91±0.334^b^	20.08±0.383^c^

Values bearing different superscripts in upper case letters in a row differ significantly at 5% (p<0.05) and in lower case letters in a row differ significantly at 1% (p≤0.01). SE=Standard error

## Conclusion

It is concluded that spermatozoa viability, motility, HOS response and post-thaw incubation response were better in Group II, in which incubation was carried out for 10 min. Furthermore, even after 30 min of post-thaw incubation, Group II (10 min) retained about 50% motile and 55% viable spermatozoa than other groups.

## Authors’ Contributions

SAL planned and performed research work for his MVSc thesis program in collaboration with guide (JKP). Author is thankful SKG and GKD for providing lab facility during his research work. RK and BB helped in collection of sample and estimation of parameters during his research work. MRV helped in statistical analysis of data. All authors read and approved the final manuscript.
